# A Game-Based Mechatronic Device for Digital Rehabilitation of Hand Function After a Stroke: Design, Prototyping, and Feasibility Study

**DOI:** 10.2196/67779

**Published:** 2025-03-19

**Authors:** Anuprita Kanitkar, Nariman Sepehri, Ariel Lezen, Sanjay Tejraj Parmar, Cherry Kit-Fong Hin, Tony Joseph Szturm

**Affiliations:** 1 Department of Physical Therapy, College of Rehabilitation Sciences, University of Manitoba, Winnipeg, MB, Canada; 2 Department of Mechanical Engineering, Price Faculty of Engineering, University of Manitoba, Winnipeg, MB, Canada; 3 SDM College of Physiotherapy, Shri Dharmasthala Manjunatheshwara University, Dharwad, India

**Keywords:** stroke, manual dexterity, hand function, poststroke, fine motor, thumb, finger, wrist, movement, motor rehabilitation, assistive technology, smart monitoring, pilot, feasibility, prototyping, prototype, nervous system, nerve, motor neuron

## Abstract

**Background:**

This paper presents an easy-to-use, affordable robotic manipulandum device (RMD) equipped with smart monitoring and assistive technologies to engage in game-based exercise and repetitive task practice. The RMD has been designed to enhance a wide range of fine motor manual dexterity skills, including thumb, finger, and wrist movements. By focusing on finger and hand functions, it extends its utility beyond basic reaching or object transfer movements. Various interchangeable 3D-printed therapy handles of different shapes and sizes can be easily attached to the RMD drive shaft. These handle movements can be used to engage with numerous affordable, commercially available computer games, allowing patients to practice tasks that involve varying movement amplitudes, speeds, precision, and cognitive challenges. Additionally, the device is capable of automatically recording and storing the patient’s real-time performance data on any given computer, integrating assessment into treatment.

**Objective:**

A pilot study was conducted with 5 patients with stroke to examine the feasibility and benefits of a 6-week game-based exercise program using the proposed device.

**Methods:**

A feasibility study was conducted with 5 participants. Data were collected using the computer game–based upper extremity assessment of manual dexterity and Wolf Motor Function Test (WMFT) before and after the intervention lasting 6 weeks.

**Results:**

The pilot study demonstrated that clients’ expectations related to manual dexterity were met. The average improvement in the functional ability score of the WMFT was 14 (SD 3) points, with all participants exceeding the minimal clinically important difference. The average reduction in total time was 30 (SD 14) seconds, with 4 of 5 participants surpassing the minimal clinically important difference. For the computer game–based upper extremity assessment, the average improvement in success rate was 23% (SD 12%), and the average decrease in response time was 105 (SD 44) milliseconds.

**Conclusions:**

Findings revealed acceptable, engaging, game-based, and task-oriented training with a high level of compliance. Substantial improvements from pre- to postintervention were observed using the WMFT and assessments of manual dexterity.

## Introduction

### Background

Upper extremity (UE) motor impairments and persistent hemiparesis commonly lead to difficulties with manual dexterity after a stroke [1]. Manual dexterity, defined as the ability to manipulate objects, is crucial for many everyday tasks, both for leisure and social interactions. These tasks often require the manipulation of objects that vary widely in physical properties and functional demands, necessitating a high degree of precision [2]. Individuals with chronic sensory-motor deficits in the UE following a stroke can greatly benefit from intensive, well-resourced therapy services [3-6]. A novel approach to enhance patient engagement in therapy is the use of computer games, which integrate various learning elements and present motor and cognitive challenges. This allows individuals to participate in focused, task-specific activities with a significant number of repetitions [7-10]. Several gaming systems have been used as rehabilitation tools [11]. Various computer input devices have been used to detect arm segments or finger motions. The corresponding motion signals are used to interact with digital avatars or objects [12,13]. However, these game-based exercise programs often fail to adequately address object handling and fine motor function–based object manipulation. Consequently, they do not account for the sensory, tactile, or proprioceptive signals from the hand that are essential for effective goal-directed object manipulation tasks. To enhance the brain’s capacity for learning, it is vital to create experiences that improve manual dexterity through guided and repetitive practice of manipulation tasks requiring precision [14-16]. Some game-based rehabilitation systems use handles or joysticks as controllers [17,18], where the handle is manipulated using wrist, elbow, and shoulder motions. However, these systems include only a few custom-made games.

To extend these systems, a cost-effective computer-based gaming platform has already been developed, which integrates various object manipulation tasks with engaging computer game activities. This platform uses a miniature, wireless, inertial-based (IB) computer mouse that directly connects object manipulation with digital gaming [19-22]. The IB mouse can be attached to a wide range of objects with different shapes, sizes, and weights and can be handled using 2-finger, 3-finger, or whole-hand motions as well as wrist, elbow, and shoulder movements. These object manipulation tasks are used to practice diverse, goal-oriented manual dexterity skills while users engage with entertaining computer games. However, this gaming system does not provide movement assistance for patients with limited active range of motion or poor movement control.

Numerous studies have assessed the feasibility and impact of various robotic systems aimed at improving UE functions in patients with stroke [23-30]. Augmented reality game–based devices focus on enhancing the range of motion in the shoulder, elbow, and wrist. However, these devices are not able to detect hand and finger movements with the required amount of precision. The camera-motion and sensor-based devices cannot detect movement with real-life objects. Thus, these devices can only detect active gross movements, neglecting object manipulation. A few robotic devices such as soft or hard gloves and exoskeletons do assist with finger and thumb flexion-extension; however, they primarily feature custom software applications that involve activities performed in digital settings rather than real object manipulations.

Given the above considerations, a low-cost, portable, multipurpose robotic manipulandum device (RMD) equipped with smart monitoring and assistive technologies for game-based rehabilitation of manual dexterity was developed. The RMD functions as a responsive, high-resolution computer mouse. In this paper, we first describe the RMD hardware and gaming software, its functionality, and related applications to provide both treatment and assessment of recovery programs targeting the manual dexterity of people after a stroke. The objective of this study is to present the results of a proof-of-principle pilot study conducted on 5 patients with stroke to examine the feasibility and benefits of a 6-week game-based exercise program using the RMD. The RMD described in this paper explains an integrated controller to generate forces that can aid voluntary movements necessary during gaming exercises, making it suitable for patients with limited movement control and those with a restricted active range of motion.

### Description of the RMD and Software

**Figure 1.**
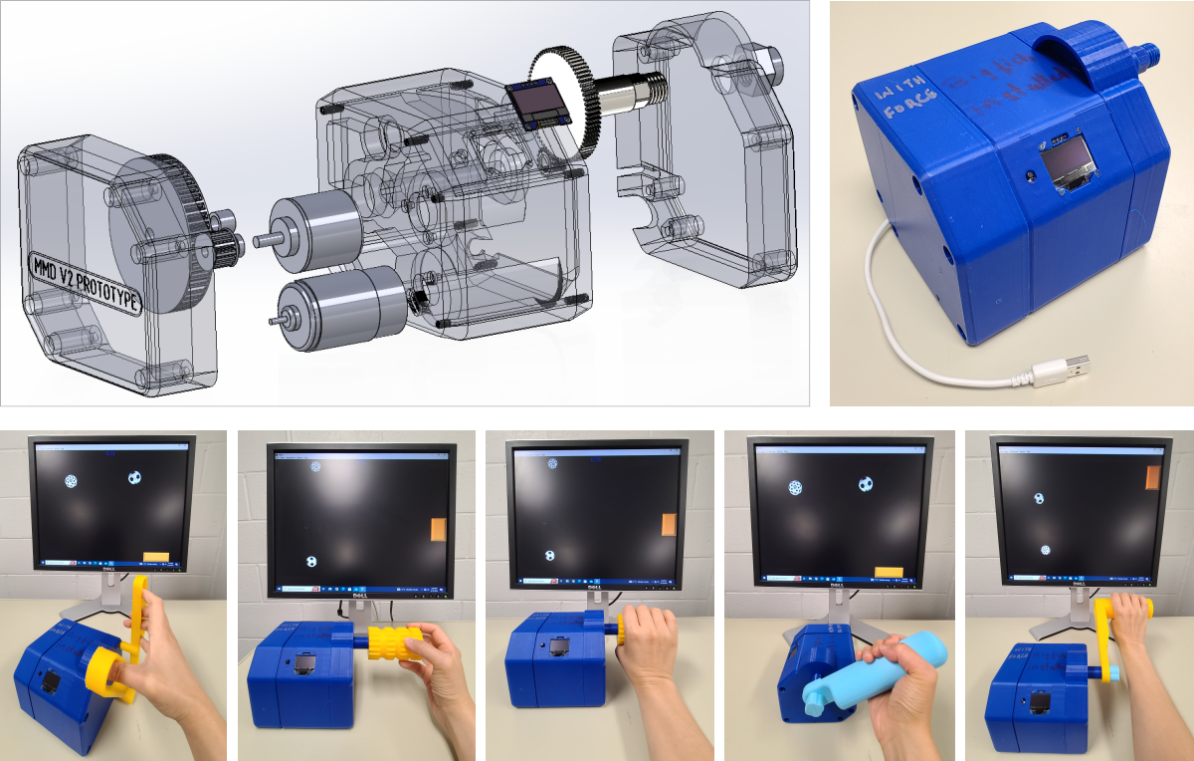
General view of the robotic manipulandum device and examples of various handles used for game-based rehabilitation of manual dexterity.

Referring to Figure 1, the RMD features a compact, integrated 3D-printed chassis that contains the interface board, actuator, sensors, power train, and rotary drive shaft. Various 3D-printed therapy handles of different shapes and sizes can be attached to the shaft. These handles are designed to help users practice a wide range of manual dexterity skills involving thumb and finger movements as well as wrist, elbow, and shoulder functions. The RMD connects to a computer using a standard USB cable. An optical encoder tracks the shaft rotation, which corresponds to the movements of the handle and controls the motion of a computer cursor or game sprite in any single-axis computer game. In this context, the rotation of the shaft is mapped to pixel coordinates on the screen. An Arduino Leonardo microprocessor manages the RMD and its interaction with the games. Additionally, the RMD features a 3-cm LED display that shows several adjustable control parameters, which the user can modify:

- Gameplay orientation: mouse horizontal or vertical motion. Many common and modern video games are played with horizontal game sprite motion, but some require vertical motion.
- Working range: users can select an active range of motion for exercises, for example, from wrist neutral to 10, 20, or 30 degrees of extension or flexion ranges of motion, depending on individual patient needs, and map this to the full-screen mouse position.
- Mouse sensitivity: this setting determines the amount of movement required to navigate the mouse across the entire display range.
- Force: the RMD is designed to facilitate various assistive and resistive movement patterns. One of its applications involves a unidirectional force field mode, where a consistent force is exerted on the output shaft in a specific direction, with both the magnitude and direction adjustable. Many patients exhibit greater impairments in finger and wrist movement in 1 direction (eg, wrist extension), making the assistance of a constant force beneficial. Conversely, the opposite movement (eg, wrist flexion) can be met with a resistive force. This context-sensitive assistive or resistive mode can enhance even minimal voluntary movements in severely affected individuals, creating opportunities for progressive exercise that increases movement demands.

Since the RMD operates as a USB plug-and-play computer mouse, it is compatible with digitally any commercially available computer game. The inclusion of gaming elements motivates patients, providing an enjoyable way to engage in repetitive movements that are often necessary for rehabilitation. The therapeutic benefits from the types of object manipulation tasks involved vary in physical and anatomical requirements. The selected computer games provide graded responses in movement amplitude, speed, and precision. Table 1 outlines several common computer games that have been extensively tested with the RMD among patients of various ages. Additionally, a specially designed rehabilitation repetitive task practice (RTP) game has been created by the University of Manitoba and validated [31-33]. This simple game records the movements of the computer mouse curser or game paddle to assess the quality of movements. This game automatically tracks patients’ goal-directed object manipulation tasks during both local and remote game–based therapy sessions, allowing for performance quantification in each session. This feedback can provide immediate results to the patient and help clinicians monitor progress over time. In practice, RMD-assisted exercises would initially use the RTP game software for therapeutic purposes. The RTP software is customizable, enabling adjustments to all game elements to suit the skill levels of patients with varying degrees of sensory-motor impairments.

**Table 1.** Big Fish Games were used in this study [34].

Game	Axis play	Start difficulty	Response time	Clicker	Precision	Distractor	Type or activity
Abundante	Horizontal	Moderate	Self-paced (time limited)	Yes	Moderate	No	Color matching by directional aiming
Action Ball	Horizontal	Moderate	Fast	Yes	Moderate	Yes	Brick buster
Aqua Ball	Horizontal	Easy	Moderate	Yes	Moderate	Yes	Brick buster
Astro Bugz Revenge	Horizontal	Moderate	Slow	Yes	Moderate	No	Color matching by directional aiming
Birds Town	Horizontal	Moderate	Moderate	Yes	Moderate	No	Color matching by directional aiming
Brave Piglet	Vertical	Easy	Fast	Yes	Low	Yes	Shooting
Bricks of Egypt	Horizontal	Moderate	Fast	Yes	Moderate	Yes	Brick buster
Butterfly Escape	Horizontal	Moderate	Moderate	Yes	Moderate	No	Color matching by directional aiming
Egyptian Ball	Horizontal	Moderate	Fast	Yes	Moderate	Yes	Brick buster
Invadazoid	Horizontal	Moderate	Fast	Yes	Moderate	Yes	Brick buster
Jar of Marbles	Horizontal	Easy	Self-paced (time unlimited)	Yes	Moderate	No	Color matching by directional aiming
Jet Jumper	Horizontal	Difficult	Fast	Yes	High	Yes	Steering and jumping
Luxor HD	Horizontal	Moderate	Moderate	Yes	Moderate	No	Color matching by directional aiming
Ricochet Recharge	Horizontal	Moderate	Fast	Yes	Moderate	Yes	Brick buster

a Matching and shooting games require participants to use a small wireless optical computer mouse, pressing the left mouse button when needed. Precision is determined by the size of the paddle and the size of the target objects. Difficulty levels include game speed, the number of distractors, and matching choices.

**Figure 2.**
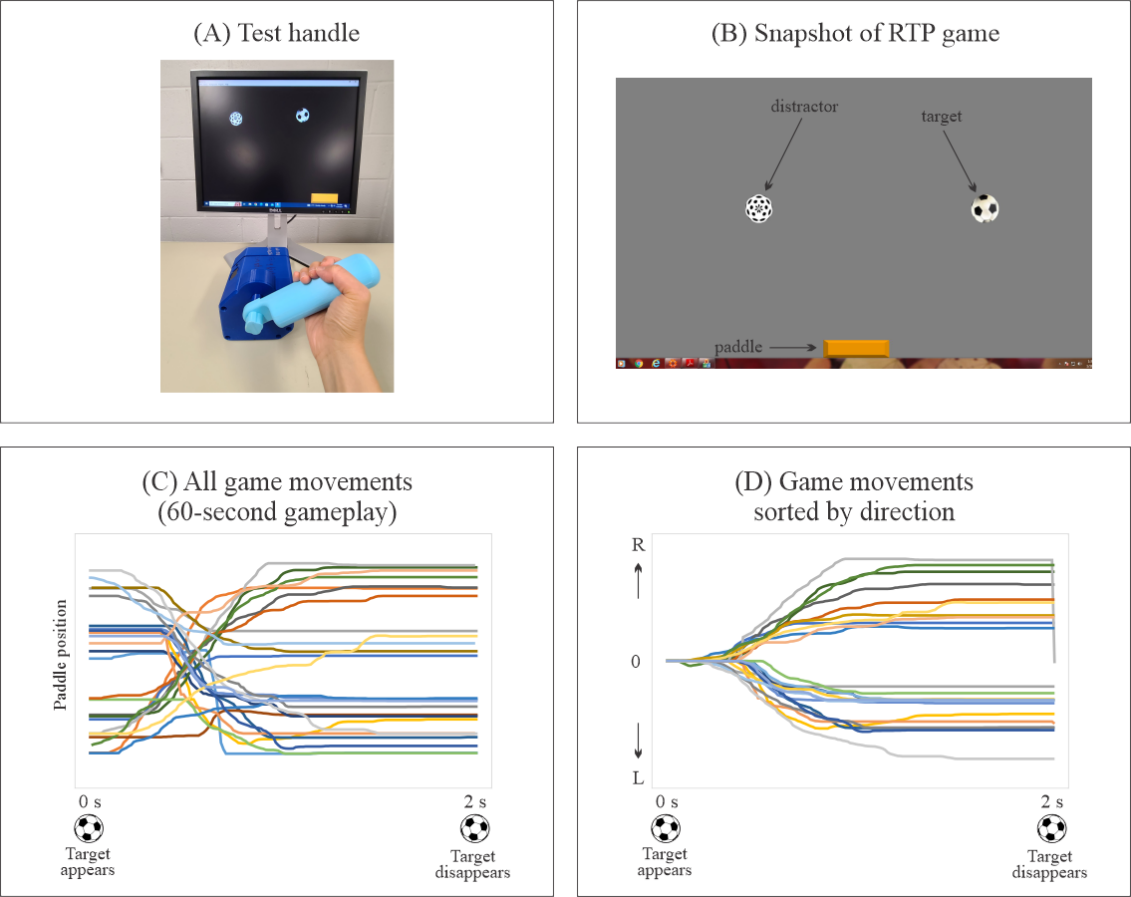
Illustration of RTP game software using the robotic manipulandum device. Panel (A) shows a healthy adult rotating a handle to move the game “paddle” and catch the “target” object while avoiding the “distractor” object. Panel (B) shows a screenshot of the game, where target and distractor objects appear at the top of the display and move to the bottom. Note that the addition of distractors is optional. Panel (C) presents single-game movement trajectories (game paddle coordinates) for all game movement responses in one session. In this example, each game event takes 2 seconds (from target appearance to disappearance), and the game is played for 60 seconds. The location of each successive target appearance is randomized. Approximately half of the 30 game events occur in each direction (leftward or rightward). Panel (D) presents overlay plots of the segmented and sorted game movement trajectories for all 30 game events; upward traces indicate leftward game movements, and downward traces indicate rightward game movements. RTP: repetitive task practice.

Figure 2 illustrates a snapshot of the RTP game, highlighting game movement responses when using the RMD. Game objects appear randomly at the top of the display, moving at unpredictable speeds and directions toward the bottom. Players aim to maneuver the game paddle to catch these moving targets, with the RMD handle rotation controlling the paddle’s motion. Distractor objects are included to increase challenge and can be toggled on or off. Configurable features include movement speed, precision (eg, sizes of game objects and paddles), movement amplitude, and the incorporation of distractors to assess the interplay between motor and cognitive processing as well as dual-task interference effects. Throughout gameplay, the RTP software logs the timing of each game object’s appearance and disappearance, defining game events, along with tracking the position of the paddle and other game objects to establish movement context. Panels C and D in Figure 2 demonstrate typical movement trajectories within the game. Various performance metrics can be captured using the RTP game, offering immediate feedback for both patients and therapists. Additionally, electronic outcome measures are recorded to monitor progress and dose-response relationships in specific exercise programs over time, including success rates (SR), response times, movement durations, accuracy, and movement variability.

It is important to note that with standard commercial games, automatic performance logging is typically unavailable. Therefore, for any training sessions—particularly those conducted at home or remotely—the RTP game developed in-house serves as a valuable resource, providing automated monitoring and quantification of players’ motor skills while engaging in a range of game-based exercises for hand and arm coordination (also referred to as telemonitoring).

**Figure 3.**
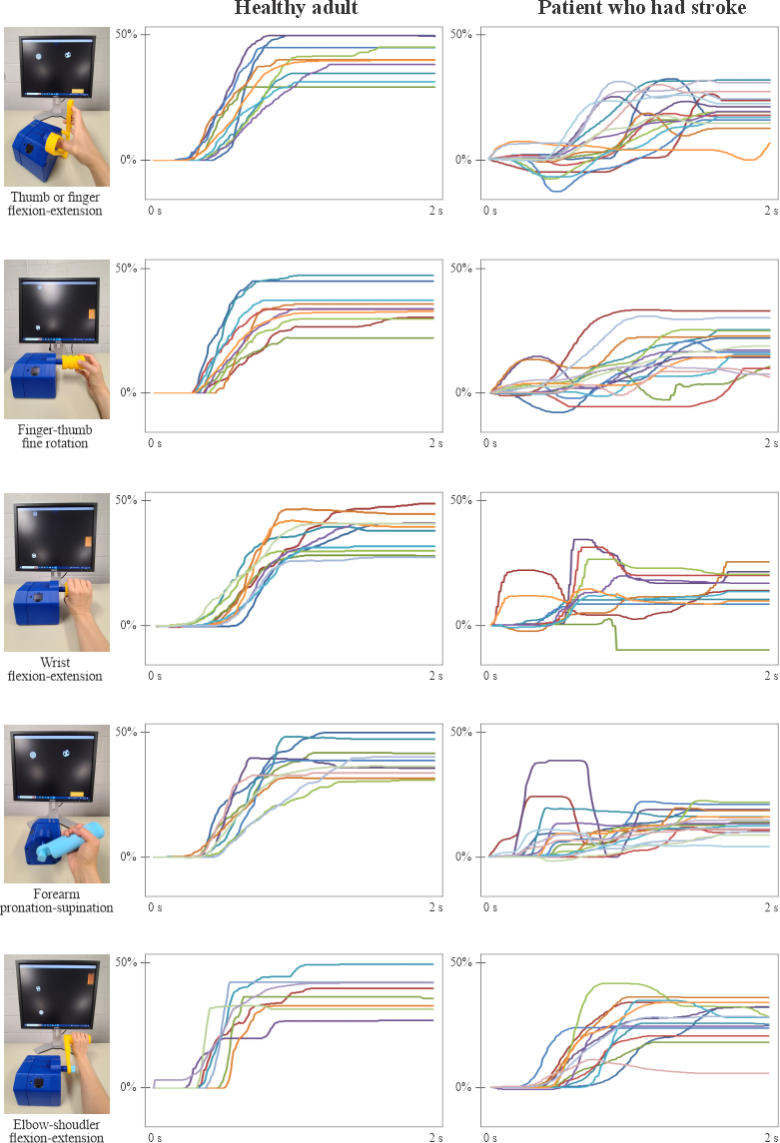
Repetitive task practice game movement trajectories of an able-bodied adult and a patient with stroke using various robotic manipulandum device handles, as described in Figure 2. The plots show segmented and sorted game movement responses for 1 direction of movement. The y-axis represents movement amplitude as a percentage of screen width (0% to 50%).

Figure 3 presents game movement trajectories of a representative able-bodied adult and a patient with stroke playing the RTP game using various handles. As can be seen in the plots, the trajectories of the 5 different manipulation tasks are similar. The SR was 100% for all manipulation tasks. For the patient with stroke, the SR ranged from 50% (thumb-finger flexion-extension) to 80% (elbow flexion-extension). Movement consistency among the 10‐12 game movement responses of the able-bodied adult was similar, as was movement onset time (MOT). Many of the movement trajectories of the patient with stroke were not smooth, exhibited small amplitudes, and demonstrated several target overshoots. It is also evident that the MOT is delayed in the participant with stroke compared to the able-bodied participant. It is, therefore, seen that the platform presented here is functional and can produce meaningful data for further analysis and treatment decisions.

Another key feature of the RMD and the associated RTP game software is that it is designed for use at home (telerehabilitation), not just in clinics. At present, the cost of the device is estimated to be less than US $70. The software automatically collects various objective outcome measures to monitor a patient’s ongoing progress instantaneously and can be traced over a period of time. These data support the development of sustainable, individualized, long-term rehabilitation protocols. Furthermore, clinical support for home and remote outreach programs can facilitate the creation of more targeted and personalized solutions for patients.

The objective of this pilot study was to evaluate the implementation, usability, acceptability, and benefits of the game-based exercise program using the developed RMD presented in this paper. The experience of participants with stroke who completed a 6-week game-based exercise program was first assessed with semistructured interviews. Interviews were conducted to investigate participants’ perspectives and opinions about expectations, acceptability, challenges, and benefits of the game-based exercise program for UE rehabilitation. Quantitative analysis pre- to postintervention was conducted next, which included the Wolf Motor Function Test (WMFT) and a computerized performance-based assessment of manual dexterity.

## Methods

### Recruitment

Participants were recruited at the clinical rehabilitation research facility of the University of Manitoba. In total, 5 individuals who had a single stroke (onset between 6 months and 5 years) and were aged 40 to 70 years participated in the study. All participants had adequate vision to see images on a standard computer monitor. Exclusion criteria were (1) excessive spasticity of the fingers and wrist (grade 2 and above on the Modified Ashworth Scale [35], (2) significant cognitive impairment (Montreal Cognitive Assessment scores less than 25 [36], and (3) any other neurological disorder except a single stroke before testing.

### Ethical Considerations

The University of Manitoba Ethics Board reviewed and approved the study (approval HS25163), and all participants provided informed consent. The consent process ensured participants comprehended the study’s objectives, procedures, potential risks, benefits, and their right to discontinue at any time. To maintain participant anonymity, all collected data were anonymized and stored in a secure, locked location. No compensation was provided, and no photographic or video recordings of participants were taken.

### Exercise Program

Participants attended 12 treatment sessions twice a week for 6 weeks. Each session lasted 45 minutes. As shown in Figures1  3, a variety of 3D-printed “therapy” handles of different shapes and sizes were used. They were designed to practice a broad range of manual dexterity skills. The exercise programs were established based on the participants’ personal goals, the degree of their hemiparesis, and functional status. A typical session involved exercise with 4 to 5 different handles, several computer games, and assistive forces of different magnitudes. Each handle-game-force combination was practiced for 2- to 3-minute intervals and repeated 2 to 3 times. Different handles required different modes of manipulation. Game movement responses were produced by thumb, finger, wrist, or elbow movements. Task demands were adjusted by changing the mouse sensitivity movement range and by adding assistive-resistive forces. Additionally, different games were selected to adjust movement speed and precision. Most participants were competitive and became frustrated if they were not successful in gameplay. Therefore, the difficulty level (movement amplitude, speed, and precision) was adjusted for all combinations of handles, game settings, and game types so that participants were successful in gameplay for at least 60% of the game events or activities. Table 1 presents a list of common computer games used in this study. Big Fish Games are selected based on the level of difficulty participants reported and their personal likes and dislikes. The choices of games presented to them were based on columns 3 to 7 of Table 1. Games with an easy level of difficulty (based on the level of precision required, the presence of distractors, and the type of executive functions required) were introduced before the moderate and difficult games. Task difficulty was also adjusted by increasing the assistive and resistive forces applied to the RMD handles.

The exercises and choice of games were updated on a regular basis, based on the participants’ improvements and personal preferences for game selection. Numerous affordable and readily accessible computer video games offer therapeutic benefits. For instance, computer games downloaded from Big Fish Games feature hundreds of arcade-style games across various genres (Table 1). Many of these games align well with the game-based RMD exercise program. In addition to requiring speed and accuracy, these games incorporate several cognitive elements, such as speed versus accuracy dynamics, distractor objects, and object-matching activities. The commercial computer games used in this pilot study are listed in Table 1. The wide variety of games ensures that the individual preferences of participants can be fulfilled. Regularly introducing new games and increasing the difficulty levels can help maintain the challenge, providing the psychological feedback necessary to keep participants engaged and motivated.

### Qualitative Analysis

At the end of the 6-week exercise program, all participants were invited to participate in an interview. They were asked a series of open-ended questions, and their responses were documented: (1) when you agreed to participate, how did you hope you would benefit from the therapy program? (2) Were there things about the game or exercise program you liked and things you did not like? (3) What did you think about the computer games that you were asked to play? Did you enjoy the game? Were there games that you did not enjoy? (4) Did you feel that this therapy program helped you? (5) If you were provided with the right settings, would you continue with these exercises?

The duration of the interviews varied among the 5 participants, lasting between 20 and 30 minutes. Participants were invited to share their thoughts, ideas, opinions, and personal experiences in detail. The analytical framework of interpretive description was used for thematic analysis [37]. All interviews were recorded, and the interviewer’s notes and comments were added to the transcriptions separately for triangulation purposes. One researcher (AK) reviewed the translated transcripts and created a coding system, while a second researcher (TJS) oversaw the process and added any additional codes for credibility purposes. A second researcher (TJS) then examined the coded data to identify any unique responses.

The content of each interview was analyzed by paraphrasing, generalizing, and abstracting. A continuous iterative process was maintained until no new themes emerged from the data. The 2 researchers then compared their analyses and resolved any disagreements in a final coding system organized into final themes and subthemes.

### Quantitative Analysis

#### Overview

The following outcome measures were obtained before and after the intervention of the 6-week exercise program:

1. Quantitative assessments of UE motor ability were conducted using the WMFT [38,39]. Participants were instructed to complete the 15 tasks of the WMFT within a 120-second time limit, and the time taken to complete each task was recorded. Additionally, the quality of movement for each task was evaluated using an ordinal scale ranging from 0 to 5, where 0 indicates no performance and 5 indicates normal movement. The final WMFT scores were the total time taken for the 15 tasks and the summed movement quality grades of the 15 tasks.
2. The RTP game was used to guide and evaluate different object manipulation tasks. In this application, several test objects with different physical properties and anatomical demands were instrumented with a wireless IB mouse. The rotation of each test object (ie, the instantaneous angular position of the IB mouse) controlled the motion of the game paddle. For a detailed description of the assessment tool, see references [20,33]. All tasks required precision in object manipulation using the finger-thumb or hand palmar surface.

#### Test Objects

**Figure 4.**
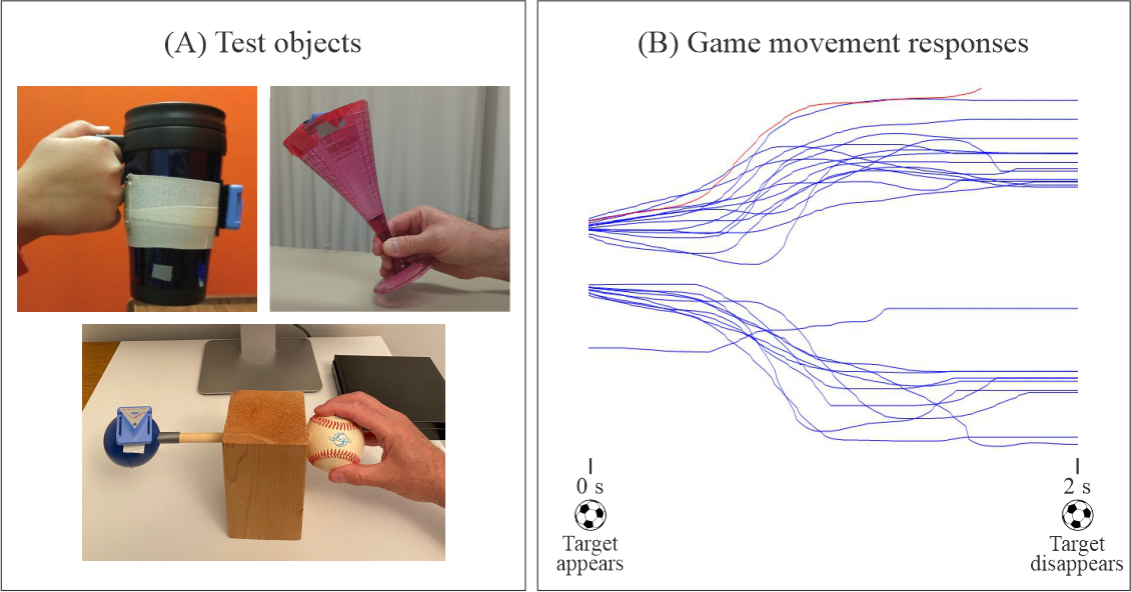
Illustration of the computer game–based upper extremity assessment tool. (A) Three test objects, each equipped with an inertial-based mouse, were used to control the repetitive task practice game paddle movements. (B) Example overlay plots of the segmented and sorted game movement responses for both movement directions: pronation-supination using a coffee mug, ulnar-radial deviation using a wine glass, and leftward-rightward rotation using a tennis ball.

In the context of the RTP game assessment, the following test object manipulation tasks were evaluated, as illustrated in Figure 4: (1) participants grasped a coffee mug to move it with concentric pronation and eccentric supination. (2) Participants held a wine glass between the thumb, index, and middle fingers. It was rotated forward and backward using radial and ulnar deviation. (3) Participants grasped a tennis ball with the thumb and fingertips tethered to a wooden block via a wooden dowel to eliminate the gravity effect. This task required the participant to rotate the tennis ball left and right.

The assessment presented here allows one to determine if there is a transfer of improvement in manual dexterity with objects used in daily life. Moderate to high test-retest reliability of the assessment tool has been reported in a group of 30 patients with stroke [20] and a group of 35 children with cerebral palsy [21].

#### Test Protocol

Participants were seated with test objects positioned within a comfortable reaching distance on an adjustable-height table. A 50-cm wide computer monitor was placed 1 m in front of them at eye level to perform the assessment game tasks. Participants received a demonstration of the game tasks and were allowed to practice trials using their unaffected arms. Figure 4 presents typical overlay plots of game movement trajectories for both movement directions for 1 game session.

#### Outcome Measures

The following outcome measures were derived from the recorded game data of the assessments: SR and average MOT. The percentage of the total number of target objects caught in 1 game trial is the SR. The time from target appearance to the start of the game paddle movement is the average MOT. MOT values are determined for each game movement response. The average is then computed over the group of game movement responses for each direction.

## Results

### Participants

Table 2 presents the demographic and clinical data of the 5 participants. All participants, who experienced a single stroke, agreed to take part in the study and provided informed consent. They were all right-handed and fully completed the 6-week program, which included 2 exercise sessions per week, each lasting at least 45 minutes.

**Table 2.** Demographic and clinical characteristics of participants.

Participant	Age (years)	Sex	Type of stroke	Duration (months)	Affected side	Hand dominance
Participant 1	67	Male	Ischemic	24	Left	Right
Participant 2	68	Male	Ischemic	16	Left	Right
Participant 3	57	Male	Ischemic	12	Left	Right
Participant 4	43	Female	Ischemic	4	Left	Right
Participant 5	51	Male	Hemorrhagic	56	Left	Right

### Qualitative Results

The following 4 themes capture the range of participants’ experiences and viewpoints regarding the prototyped RMD exercise program: expectations, difficulties with technology, engagement with therapy, and future expectations. Table 3 presents examples of participants’ direct quotes for each interview question (theme).

**Table 3.** Typical participant responses to interview questions.

Theme	Response
Expectations	“I get into problems while handling day-to-day things. I often have trouble gauging how much distance and pressure I need. The other day I squeezed the soda cup too hard and spilled everywhere. I am hoping to improve the finer aspects” [Participant 1]. “My consultant physician told us that I was never going to use my fingers. When we heard about this program, we thought it might help” [Participant 3].
Difficulties with technology	“Learning how to use the RMD was hard at first. Learning how to move the mouse when my arm is so restricted, you know?” [Participant 3]. “I am not a tech-savvy person. It took a while to get used to the games and the robot (RMD)” [Participant 4]. “Coming to therapy twice a week and getting a ride in winter was a lot. But the home-based therapy was not working, so we decided to do it” [Participant 5].
Engagement with therapy	“I am very competitive. I like that the computer games challenged me. It was fun” [Participant 1]. “It (conventional therapy) did not show much improvement. It did not seem like it was worth the trouble. I wanted to check out this option (computer games-based protocol) because it sounded new, something fun” [Participant 4]. “I could comb my hair again. That was something!” [Participant 2]. “My hand felt completely immobile earlier; now I can use it to support my other hand for different tasks” [Participant 3]. “I was happy to see that you guys created a steering wheel handle for me to relearn driving” [Participant 5].
Future expectations	“Honestly, I think we could have done this from home if you had enough of these (RMDs). We can just download these games on my laptop” [Participant 5].

#### Expectations

All participants indicated that the primary reason for their participation in this exercise program was to improve their hand function, particularly in handling and manipulating objects. One participant agreed to participate because his therapist recommended the program. It is noteworthy that it had been several weeks to over 3 years since the participants last received physiotherapy or occupational therapy.

#### Difficulties With Technology

In total, 3 of the 5 participants reported that they had not played computer games before. However, they noted that the games were easy to learn. All participants found it intuitive to use the RMD as a game controller. They all considered the exercise program challenging and expressed that it was difficult to play the games by manipulating the RMD handle. Nevertheless, with practice, the exercises became significantly easier. All participants exhibited competitiveness and experienced frustration when they could not successfully play the games. This was taken into account, and the games were carefully selected to match the skill levels of each participant.

#### Engagement With Therapy

All participants stated that they had previously undergone physiotherapy and occupational therapy for several weeks. They expressed appreciation for the one-to-one therapy sessions, noting that receiving immediate feedback and guidance from the therapist was very helpful. Participants also reported that it was beneficial to know which games to use and why. They indicated a preference for certain games and appreciated the variety available to them during therapy. Furthermore, all participants commented that it was more enjoyable and easier to perform game-based exercises than conventional exercises.

#### Future Expectations

All participants expressed a desire to continue the program and inquired whether it would be possible to use the device at home.

### Quantitative Results

Table 4 presents the pre- and postintervention test scores for the WMFT, highlighting the changes observed from pre- to postintervention. In patients with stroke, the minimal clinically important difference (MCID) for the functional ability score has been reported to range from 3 to 6 points, while the MCID for the total time of the WMFT is 22 seconds [40]. All 5 participants in this study demonstrated postintervention improvements that exceeded the reported MCID for the functional ability measure (with a range of improvement between 9 and 16 points). In total, 4 of the 5 participants exhibited improvements in total time that surpassed the MCID (with a range of improvement between 23 and 28 seconds).

**Table 4.** Pre- and postexercise Wolf Motor Function Test scores and magnitude of change.

Participant	Functional ability score (maximum: 75)			Total time (seconds)		
	Pre	Post	Change	Pre	Post	Change
Participant 1	19	28	9	119	71	48
Participant 2	19	34	15	118	84	34
Participant 3	13	27	14	73	62	11
Participant 4	12	28	16	104	71	33
Participant 5	9	23	14	89	65	24
Average (SD)	14 (4)	28 (4)	14 (3)	101 (20)	71 (8)	30 (14)

**Figure 5.**
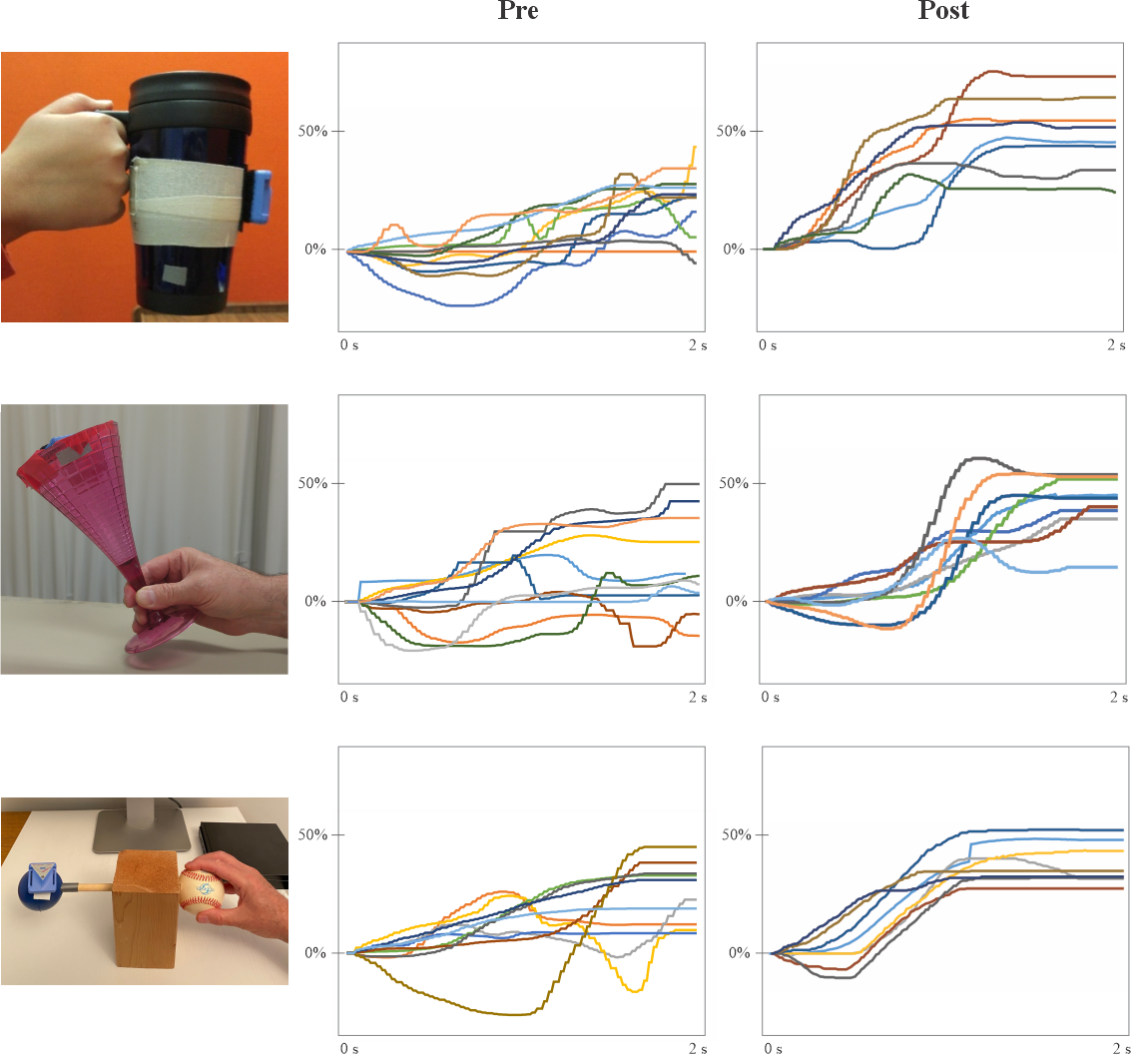
Examples of repetitive task practice game movement responses (1 direction) from different participants using the 3 assessment test objects, taken at pre- and postintervention time periods.

Figure 5 displays example plots of game movement responses using the 3 test objects, recorded at baseline and after the completion of the 6-week exercise program from different participants. Visual inspection reveals a clear improvement in movement quality, amplitude, and consistency. As indicated in Table 5, substantial improvements were observed in SR and response time for all 5 patients. For SR, the average improvement was 23% (SD 12%), while for response time, there was an average decrease of 105 (SD 44) milliseconds. It is noteworthy that typical response times were approximately 600 milliseconds.

**Table 5.** Pre- and postexercise test scores and magnitude change of object manipulation tasks for all participants^a^.

Participant and test object		Success rate (%)			Response time (milliseconds)		
		Pre	Post	Change	Pre	Post	Change
**Participant 1**							
	Object 1	42	91	49	723	530	193
	Object 2	65	89	24	478	423	55
	Object 3	51	68	17	792	697	95
	Average (SD)	53 (12)	83 (13)	30 (17)	664 (165)	550 (138)	114 (71)
**Participant 2**							
	Object 1	50	100	50	528	532	−4
	Object 2	54	62	8	780	702	78
	Object 3	33	100	67	771	421	350
	Average (SD)	46 (11)	87 (22)	42 (30)	693 (143)	552 (142)	141 (185)
**Participant 3**							
	Object 1	42	64	22	845	811	34
	Object 2	70	75	5	845	771	74
	Object 3	61	73	12	794	805	−11
	Average (SD)	58 (14)	71 (6)	13 (9)	828 (29)	796 (22)	32 (43)
**Participant 4**							
	Object 1	54	85	31	576	490	86
	Object 2	50	55	5	786.	515	271
	Object 3	66	80	14	784	725	59
	Average (SD)	57 (8)	73 (16)	17 (13)	715 (121)	577 (129)	139 (115)
**Participant 5**							
	Object 1	66	78	12	823	783	40
	Object 2	72	92	20	806	588	218
	Object 3	56	72	16	760	722	38
	Average (SD)	65 (8)	81 (10)	16 (4)	796 (33)	698 (100)	99 (103)

a Values are the average of left and right game movements. Object 1: coffee mug; object 2: wine glass; and object 3: tennis ball (Figure 5).

## Discussion

### Principal Findings

This paper introduced a rehabilitation device that provides flexible, game-based RTP targeting manual dexterity and includes means to automatically record and assess patients’ manual dexterity skills using the RTP software. The 6-week exercise program resulted in clinically significant improvement. In terms of the WMFT, on average, participants showed an improvement of 14 (SD 3) points in functional ability score and a reduction of 30 (SD 14) seconds in total time. Additionally, for the computer game–based UE assessment, the average improvement in success rate was 23% (SD 12%), while the average decrease in response time was 105 (SD 44) milliseconds. The proposed system not only addresses patients’ exercise needs but also integrates enjoyment and learning through a gaming platform.

The change in WMFT scores exceeded the MCID for all participants. The WMFT measures daily activities involving fingers, such as picking up small objects and using hand tools. Significant improvements in the WMFT were observed, even though these specific tasks were not practiced during the game-based manipulation program. The WMFT also assesses visual perceptual skills for tasks like stacking blocks and drawing figures. The RMD game tasks, which require precision movements based on visual feedback, showed substantial improvements in both the WMFT tasks and object manipulation tasks in the RTP game. Participants with stroke noted that the game-based exercises were challenging yet engaging and enjoyable.

Handles of different sizes and shapes were used to target precision, goal-directed movements of the thumb, fingers, and wrist as well as combinations of UE movements. In addition to the types of handles used, computer games also possess therapeutic value. Different commercial video games require varying levels of movement speed, accuracy, and amplitude. For example, participants with severe impairments were able to successfully play computer games when the selected games involved slow movements and low precision (ie, large game paddles and target game objects). Participants with moderate to mild impairments could engage with computer games that required faster speeds and greater precision.

The games also involved various executive cognitive functions, including visual search and spatial processing of moving targets and distractors. The diverse range of games and regular updates to difficulty levels are important for maintaining engagement and challenge.

The RMD is configured to function exactly as a plug-and-play computer mouse and, therefore, can be used to play many commercial computer games. This allows easy access to a large source of commercial games. To meet the needs of each individual, the RMD can be customized to suit specific rehabilitation needs and preferences. This adaptability allows patients to engage with a variety of gaming experiences tailored to their specific motor and cognitive rehabilitation goals. A key feature of the program is to increase the number of repetitions of goal-directed movements at varying speeds and accuracy levels. High intensity and a high number of repetitions are crucial to drive neuroplasticity and functional improvement in patients with stroke [41-45].

Each game-handle combination was played for 3 to 5 minutes, and typically, each game event took approximately 2 seconds. Therefore, participants made 90 to 150 goal-directed game movement responses during this time period. Each session lasted 45 minutes and included 7 to 8 different handles, resulting in several hundred game-handle combinations. The goal-directed game movement responses varied in amplitude, speed, and direction. During gameplay, visual feedback of the game sprite or paddle relative to the game target and distractor objects was used to initiate and guide each contextual game movement response, supporting implicit learning of eye-hand coordination. Additionally, the selected video games featured unpredictable trajectories for game target motion, promoting variable practice.

Interestingly, significant improvements were observed in a participant who was 5 years after a stroke, which was unexpected given that most studies include participants less than 2 years after a stroke. Although some studies have reported significant improvements in UE function 3 to 5 years after a stroke, this finding is based on only 1 participant. Future randomized controlled trials are needed to examine the effectiveness of game-based task-specific exercises for participants 3 to 5 years after a stroke.

Recovery programs can be extensive, involving RTP for many months. A key feature of the RMD is its design for home use (telerehabilitation). In this regard, the cost of the electronic components, motor housing, and handles is less than US $70. Additional costs, several times this amount, will likely be required for the commercialization of the RMD system. The RMD can initially be used in a supervised clinical setting and then transitioned to home use while being monitored by clinicians. The telemonitoring capabilities of the system (ie, RTP game) could allow clinicians to track changes in function and compliance, facilitating the development of sustainable and individualized programs. Prompt clinical assistance for home and remote outreach programs will foster more tailored and effective solutions for patients, facilitating the intended training outcomes. This will require further development to produce a secure content management system for individual electronic game data to be updated and stored for processing as well as to generate queries and reports for registered eHealth stakeholders (eg, therapists, physicians, and third-party insurance providers).

### Limitations

The unidirectional force mode, while assisting movement in 1 direction, results in resistance forces in the opposite direction, which may not be desirable. A real-time intelligent control scheme is under development, involving communication between the RMD software and the RTP game. In the upcoming system, the controller will receive coordinates for both the game targets and the paddle, which is controlled by handle rotations. This information about movement directions and amplitudes can then be used. The system will determine the direction and magnitude of the force necessary to rotate the handles effectively to move the game paddle within the RTP game. Notably, this closed-loop assistance can be offered in both movement directions during gameplay. This context-sensitive assistive mode helps facilitate limited voluntary movements in severely affected individuals.

### Conclusions

The results of the pilot study indicate the feasibility, acceptability, and positive outcomes of the RMD game–based system for enhancing manual dexterity in people with stroke who have moderate UE motor impairments. The intervention resulted in clinically significant improvements, with all participants showing enhanced performance in the WMFT beyond the MCID. These findings suggest that the system has the potential to advance rehabilitation treatments for finger, thumb, and wrist recovery in people with stroke. The long-term effects of this training on manual dexterity will need to be evaluated in future randomized controlled trials. However, the current findings are encouraging and provide a strong basis for further research and development.

## Abbreviations

IB: inertial-based
MCID: minimal clinically important difference
MOT: movement onset time
RMD: robotic manipulandum device
RTP: repetitive task practice
SR: success rate
UE: upper extremity
WMFT: Wolf Motor Function Test
